# Acute Necrotizing Pancreatitis With Infected Peripancreatic Collections and Upper Gastrointestinal Bleed Managed Via Multimodal Endoscopic Intervention

**DOI:** 10.7759/cureus.94874

**Published:** 2025-10-18

**Authors:** Mariam Saleem, Muhammad Rasikh, Muhammad Salman Tariq, Muhammad Subhan, Khizer Sohail, Muhammad A Basit

**Affiliations:** 1 Medicine, Al-Aleem Medical College, Lahore, PAK; 2 Internal Medicine/Gastroenterology, Jinnah Hospital, Lahore, Lahore, PAK; 3 Medicine and Surgery, Allama Iqbal Medical College, Lahore, PAK; 4 Internal Medicine, Allama Iqbal Medical College, Lahore, PAK; 5 Internal Medicine, Jinnah Hospital, Lahore, Lahore, PAK; 6 Internal Medicine, Jinnah Postgraduate Medical Centre, Karachi, PAK; 7 Internal Medicine, Quaid-e-Azam Medical College, Bahawalpur, PAK

**Keywords:** acute necrotizing pancreatitis, acute pancreatitis, cbd stenting, endoscopic necrosectomy, ercp, eus-guided drainage, lumen-apposing metal stent (lams), peripancreatic collection, severe acute pancreatitis (sap), upper gi bleed

## Abstract

Acute pancreatitis is a common but potentially life-threatening condition. Although most cases are self-limiting, severe forms such as necrotizing pancreatitis can lead to local complications like pancreatic necrosis or acute necrotic collections (with or without infection), as well as systemic complications such as gastrointestinal bleeding. Management becomes more complex when these complications coexist. A 37-year-old man with no prior comorbidities presented with epigastric pain and vomiting. After imaging, he was found to have choledocholithiasis with acute biliary pancreatitis. After endoscopic retrograde pancreatography and biliary duct stenting, the patient continued to have a fever along with the development of pain, and computerized tomography showed features of necrotizing pancreatitis with evolving fluid collections. Despite conservative treatment, he developed infected necrotic collections requiring endoscopic cystogastrostomy and placement of a lumen-apposing metal stent. A complication of upper GI bleeding occurred during the procedure. Cultures from the drained collection revealed *Klebsiella pneumoniae*, *Enterobacter*, and *Enterococcus faecium*. After three sessions of endoscopic necrosectomy, significant clinical improvement was observed. This case demonstrates a rare yet crucial clinical situation of concomitant infected necrosis and gastrointestinal hemorrhage during the procedure of endoscopic drainage, which necessitated a stepped-up multimodal approach. It underscores the importance of early multidisciplinary decision-making and endoscopic innovation in managing complex pancreatitis.

## Introduction

Acute pancreatitis (AP) is an inflammatory disorder of the pancreas that varies in severity from mild, self-limiting disease to life-threatening illness [1]. Globally, the annual incidence ranges from 13 to 45 cases per 100,000 population, with gallstones and alcohol consumption being the most common etiologies [1,2]. Approximately 80% of cases resolve without complications; however, 15-20% progress to severe acute pancreatitis (SAP), often accompanied by pancreatic necrosis, systemic inflammatory response syndrome (SIRS), and multi-organ dysfunction [3]. Infected pancreatic necrosis (IPN) occurs in 30% of cases in SAP, while mortality is almost 30% if it is not diagnosed and treated on time [4].

The treatment of infected pancreatic necrosis has been transformed by the step-up strategy, as supported by the Revised Atlanta Classification and the European Society of Gastrointestinal Endoscopy (ESGE), making endoscopic ultrasound (EUS)-guided drainage and necrosectomy first-line treatments in place of invasive surgery [5,6]. Nevertheless, the effectiveness of such an endoscopic technique is challenged severely by co-existing gastrointestinal (GI) bleeding, an unusual but potentially fatal complication [5]. The pathogenesis of GI hemorrhage here is complex, mainly resulting from pseudoaneurysm formation and rupture as a consequence of enzymatic autodigestion of peri-pancreatic arteries, the creation of gastric varices secondary to sinistral portal hypertension as a result of splenic vein thrombosis, and the formation of direct inflammatory fistulas into the gut wall [6]. In addition, the surgical process can be a causative site of excessive bleeding, from cysto-enterostomy or from vessels in the necrotic cavity when debriding [6]. Treatment of such complicated cases requires a complex, multidisciplinary protocol with initiation in resuscitation and expedited localization, often through computed tomography angiography [6,7]. While endoscopy in general can manage superficial or variceal bleeding, the definitive management of hemorrhage secondary to pseudoaneurysms or deep cavitary vessels nearly always demands angiography with transarterial embolization [8]. This leaves a significant clinical conundrum when active bleeding occurs in conjunction with uncontrolled infection, since the need to control bleeding tends to impede necessary drainage and debridement [8]. In contrast to advanced abilities, the simultaneous management of the two conditions is ill-defined in the literature, with significant uncertainty regarding the ideal timing and integration of therapeutic modalities [9,10]. We present a rare case of SAP complicated by infected peripancreatic collections and upper gastrointestinal bleeding during cystogastrostomy. In this case, endoscopic retrograde cholangiopancreatography (ERCP), lumen-apposing metal stent (LAMS) implantation, repeated endoscopic necrosectomies, and EUS-guided drainage are all examples of a successful multidisciplinary therapeutic approach. It emphasizes the significance of integrated care, early detection, and following evidence-based step-up procedures. 

## Case presentation

A 37-year-old married man with three children, who is only an occasional smoker and has no known chronic medical illnesses, came into the outpatient department on October 21, 2024, accompanied by severe, constant epigastric pain radiating to the back and followed by multiple non-bloody, non-bilious vomiting episodes for 24 hours. He denied fever, diarrhea, prior similar episodes, or any recent alcohol intake. He had no personal or family history of diabetes mellitus, hypertension, ischemic heart disease, hyperlipidemia, or pancreatitis. There was no history of recent medication use, herbal supplements, abdominal trauma, or illicit drug use. Given his demographic profile, absence of alcohol history, and the abrupt onset of pain with vomiting, the differential at presentation included biliary colic, acute cholecystitis, acute pancreatitis, and peptic ulcer disease.

On physical examination, the patient was alert, oriented, and in mild distress from pain. His vital signs at presentation were temperature at 98.4°F, blood pressure at 124/78 mmHg, heart rate at 86 beats per minute, respiratory rate at 18 per minute, and oxygen saturation at 98% on room air. Physical examination revealed only mild, localized epigastric tenderness without peritoneal signs (guarding or rebound). No masses, jaundice, ascites, or cardiopulmonary abnormalities were detected.

Initial laboratory evaluation confirmed acute pancreatitis, demonstrating a marked elevation of pancreatic enzymes (serum amylase 850 U/L, lipase 1,200 U/L). The presence of leukocytosis with a left shift (white blood cell count 18.5 x 10⁹/L with 85% neutrophils) was consistent with a significant systemic inflammatory response. Furthermore, a cholestatic pattern was evident on liver function tests, characterized by elevated alkaline phosphatase (220 U/L) and gamma-glutamyl transferase (300 U/L), accompanied by a mild hyperbilirubinemia (total bilirubin 2.2 mg/dL). This biochemical profile strongly pointed toward biliary obstruction as the underlying etiology of the pancreatitis. His renal function was normal, and the fasting lipid profile ruled out hypertriglyceridemia as a cause.

**Figure 1:**
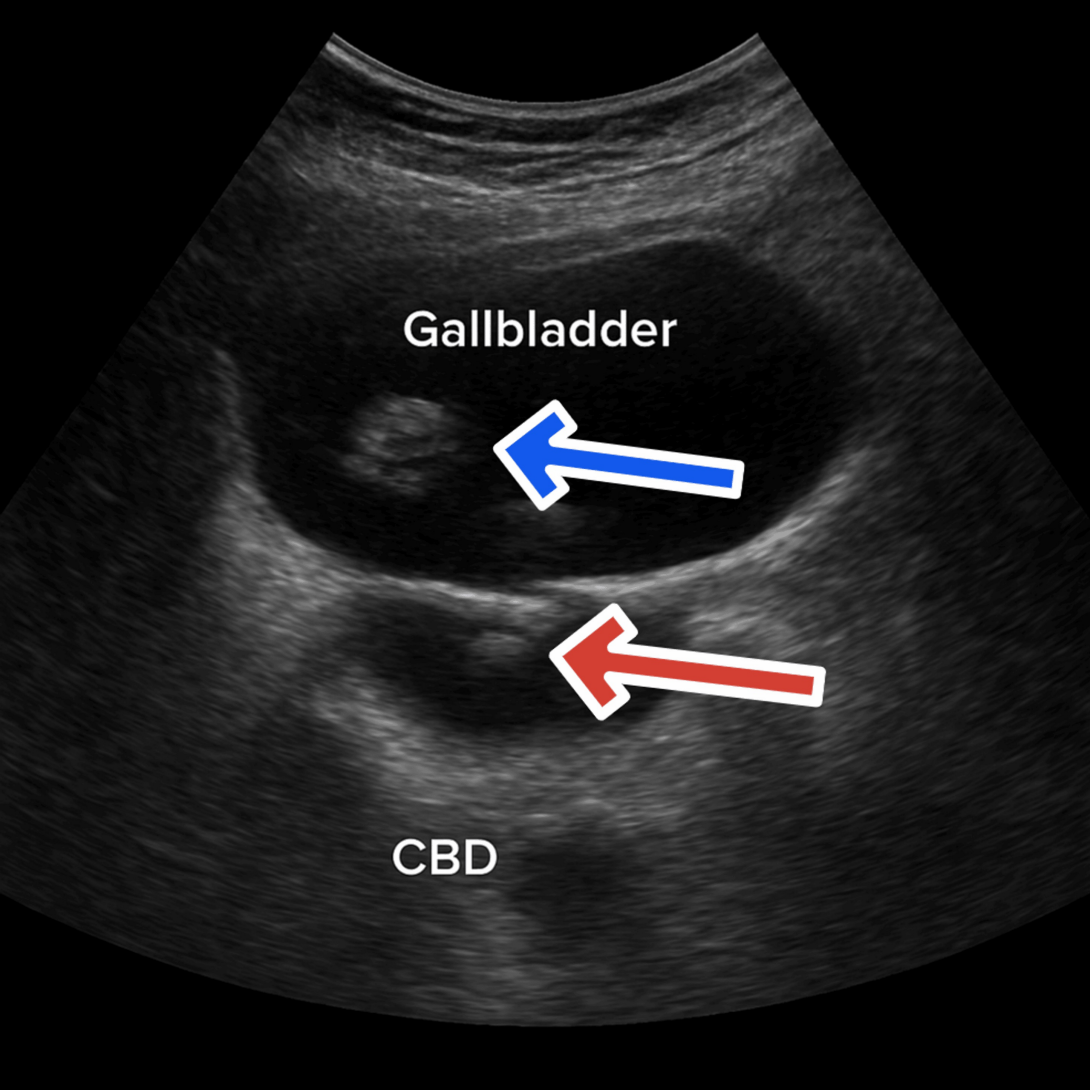
Ultrasound of the abdomen showing the distended gall bladder and common bile duct with multiple calculi (Oct 21, 2024) Abdominal ultrasound demonstrating a distended gallbladder containing multiple calculi (aggregate size up to 15 mm) with mild wall thickening as shown by the blue arrow. The common bile duct (CBD) is dilated to 12 mm, showing an echogenic focus consistent with an obstructing 6 mm CBD stone (red arrow). No pericholecystic fluid or intrahepatic biliary dilation is observed. These findings support the diagnosis of acute biliary pancreatitis secondary to choledocholithiasis.

An urgent abdominal ultrasound showed a distended gallbladder containing multiple calculi measuring up to 15 mm in aggregate and a dilated common bile duct (CBD) measuring 12 mm with an obstructing echogenic focus consistent with a 6 mm CBD stone, as shown in Figure 1.

**Figure 2:**
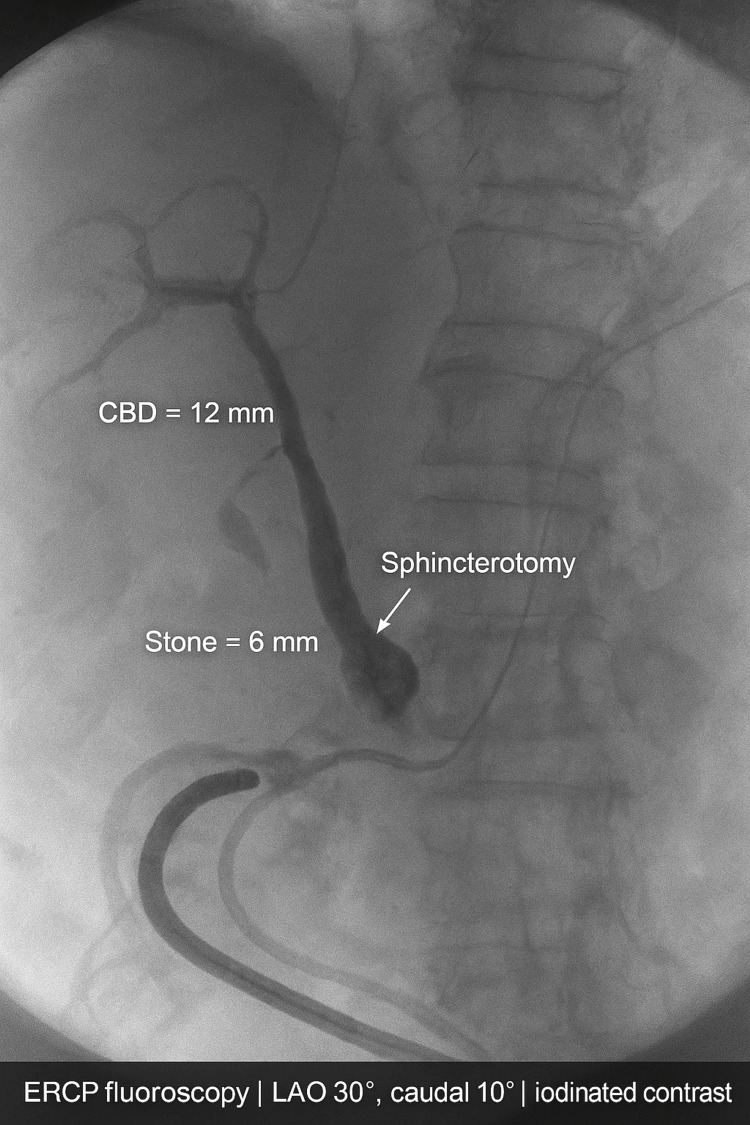
ERCP fluoroscopy showing distal CBD stone with sphincterotomy and biliary stent (Oct 23, 2024) ERCP: Endoscopic Retrograde Cholangiopancreatography, CBD: Common Bile Duct

The liver and spleen were sonographically normal, and no peripancreatic fluid collections were visualized at this stage. Given the obstructive CBD stone in the setting of acute biliary pancreatitis, an urgent ERCP was performed on the same day. Cannulation was achieved, followed by sphincterotomy and balloon extraction of the CBD stone, resulting in satisfactory ductal clearance. A 7 Fr × 10 cm plastic biliary stent was then inserted to ensure continued bile drainage and prevent recurrent obstruction, as shown in Figure 2.

**Figure 3:**
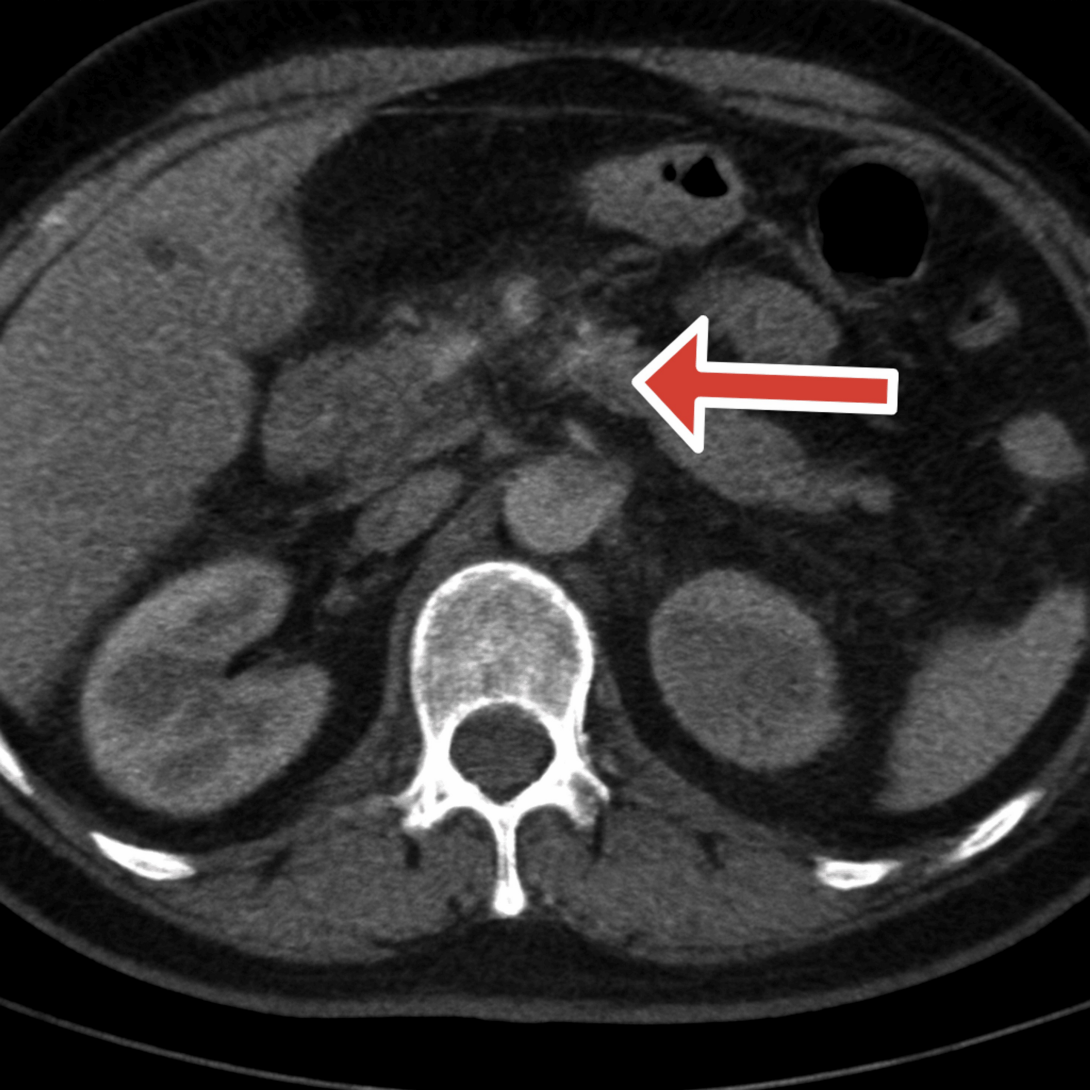
Contrast-enhanced CT of the abdomen and pelvis showing necrotic pancreatic parenchyma (Oct 24, 2024) CT: Computerized tomography The red arrow shows a necrotizing pancreas with mild fluid. Modified CTSI (Computed Tomography Severity Index): Pancreatic inflammation: Intrinsic pancreatic abnormalities with peripancreatic inflammation (Score: 2). Pancreatic necrosis: Approximately 30–50% non-enhancing parenchyma (Score: 4). Extrapancreatic complications: Presence of peripancreatic fluid collections (Score: 2). Total Modified CTSI Score: 8/10. Severity Grade: Severe Acute Necrotizing Pancreatitis

The common bile duct (CBD) stent was placed following stone clearance, consistent with standard practice in the setting of concomitant cholelithiasis. This strategy aims to prevent recurrent biliary complications, such as pancreatitis or cholangitis, which can occur from residual sludge, microcalculi, or papillary edema in the interval before definitive cholecystectomy. Within 24 hours post-ERCP, the patient developed a high-grade fever (peaking at 106°F/41.1°C) accompanied by rigors. This febrile response was refractory to aggressive intravenous crystalloid resuscitation (Lactated Ringer's solution), broad-spectrum intravenous antibiotics (piperacillin-tazobactam 4.5 g every 8 hours), and analgesic therapy with intravenous tramadol. Despite biliary decompression with stent placement, the patient’s condition deteriorated over the following days, raising concern for post-ERCP pancreatitis or procedure-related exacerbation. A contrast-enhanced CT abdomen on October 24, 2024, demonstrated diffuse pancreatic enlargement with patchy non-enhancing areas consistent with necrosis and a small peripancreatic necrotic collection near the pancreatic tail, as shown in Figure 3.

**Figure 4:**
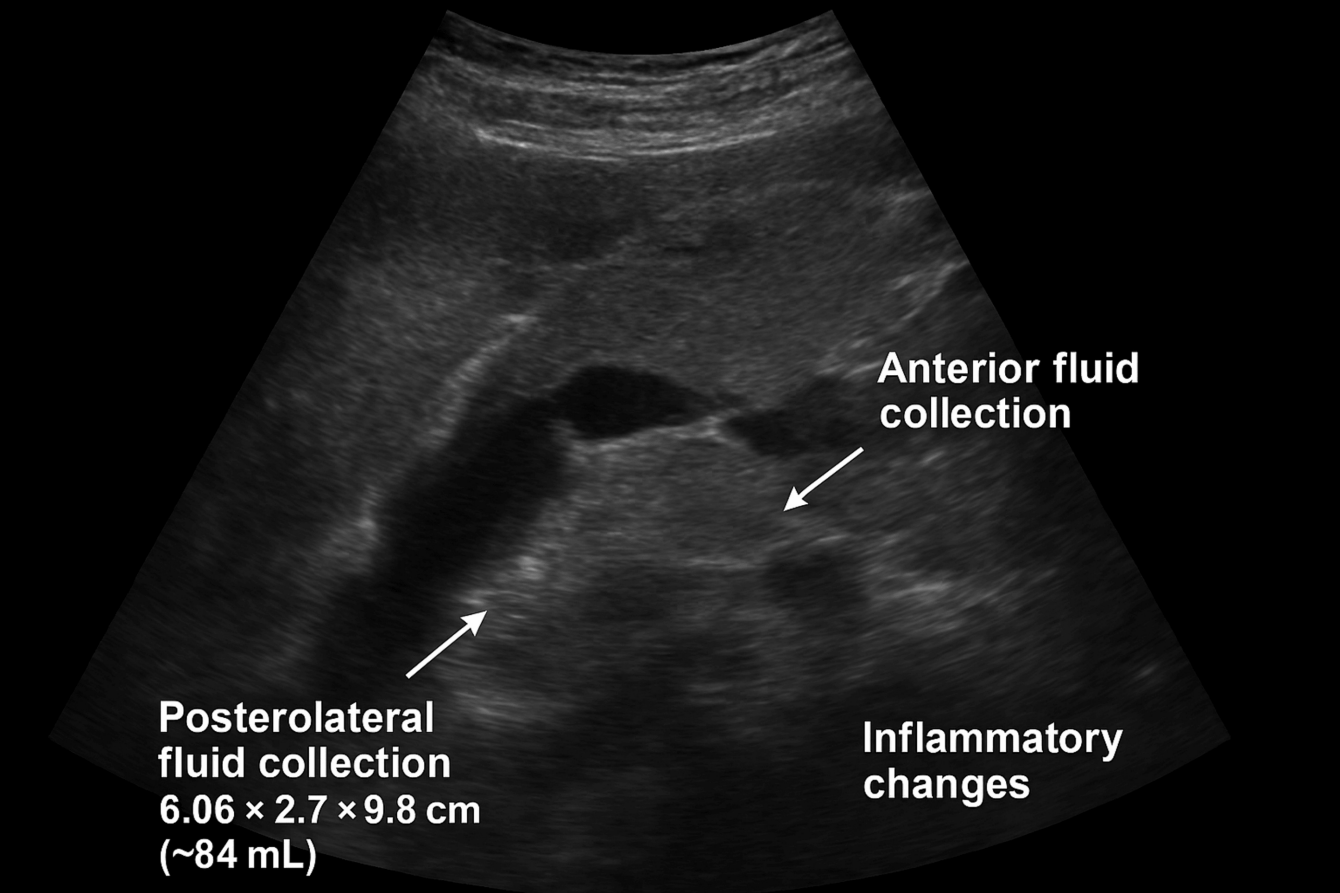
Abdominal ultrasound showing inflammatory changes in pancreatic parenchyma with fluid collections (Nov 04, 2024)

The Modified CT Severity Index score was 8, confirming a diagnosis of severe acute pancreatitis. The clinical picture was consistent with severe necrotizing biliary pancreatitis, now complicated by suspected early infected pancreatic necrosis. This critical distinction between a sterile systemic inflammatory response syndrome (SIRS) and true infection was supported by marked inflammatory markers, including a C-reactive protein (CRP) of 285 mg/L, a procalcitonin level of 4.5 ng/mL, and a leukocytosis of 18.5 x 10⁹/L with 90% neutrophils. The persistence of a high-grade fever and clinical deterioration despite broad-spectrum antibiotics (piperacillin-tazobactam) further heightened the suspicion for infected necrosis over a purely inflammatory state. The patient was managed in a monitored inpatient setting with continued intravenous hydration, bowel rest, parenteral nutrition initiation, and escalation of antibiotics to cover gram-negative and anaerobic organisms commonly implicated in infected pancreatic necrosis. Serial clinical and laboratory assessments were performed to monitor organ dysfunction. His abdominal pain gradually localized over the following 10 days, and inflammatory markers remained elevated. A repeat abdominal ultrasound on November 4, 2024, as shown in Figure 4, demonstrated two evolving peripancreatic collections consistent with acute necrotic collections (ANCs) as per the Revised Atlanta Classification. These included one collection anterior to the pancreatic body measuring 6.06 × 2.7 × 9.8 cm (~84 mL) and another posterolateral to the pancreatic tail measuring 9.9 × 4.29 cm, both with significant surrounding inflammatory changes. The increase in collection size, coupled with persistent clinical features of ongoing sepsis, prompted a multidisciplinary discussion involving gastroenterology, interventional endoscopy, and surgery.

A multidisciplinary consensus was reached to proceed with endoscopic drainage, given the radiographic maturation of the collections and strong clinical evidence of infected necrosis. On November 5, 2024 (approximately 15 days after symptom onset on October 21), an EUS-guided cystogastrostomy was performed with deployment of a lumen-apposing metal stent (LAMS). The timing of intervention, though prior to the traditional four-week mark, was necessitated by the patient's refractory sepsis and clinical decline despite maximal medical therapy. The procedure was complicated by an acute upper gastrointestinal hemorrhage. The bleeding was a direct iatrogenic complication of the cystogastrostomy, originating from the transmural tract. Hemostasis was achieved endoscopically using a combination of hemostatic spray and through-the-scope hemoclips. Microbiological cultures of the aspirated necrotic material grew *Klebsiella pneumoniae*, *Enterobacter* species, and *Enterococcus faecium*. No anaerobic organisms were isolated; the infection was therefore defined as polymicrobial based on the presence of multiple, distinct aerobic bacterial species. Intravenous antibiotics were subsequently tailored according to the antimicrobial susceptibility profile.

**Figure 5:**
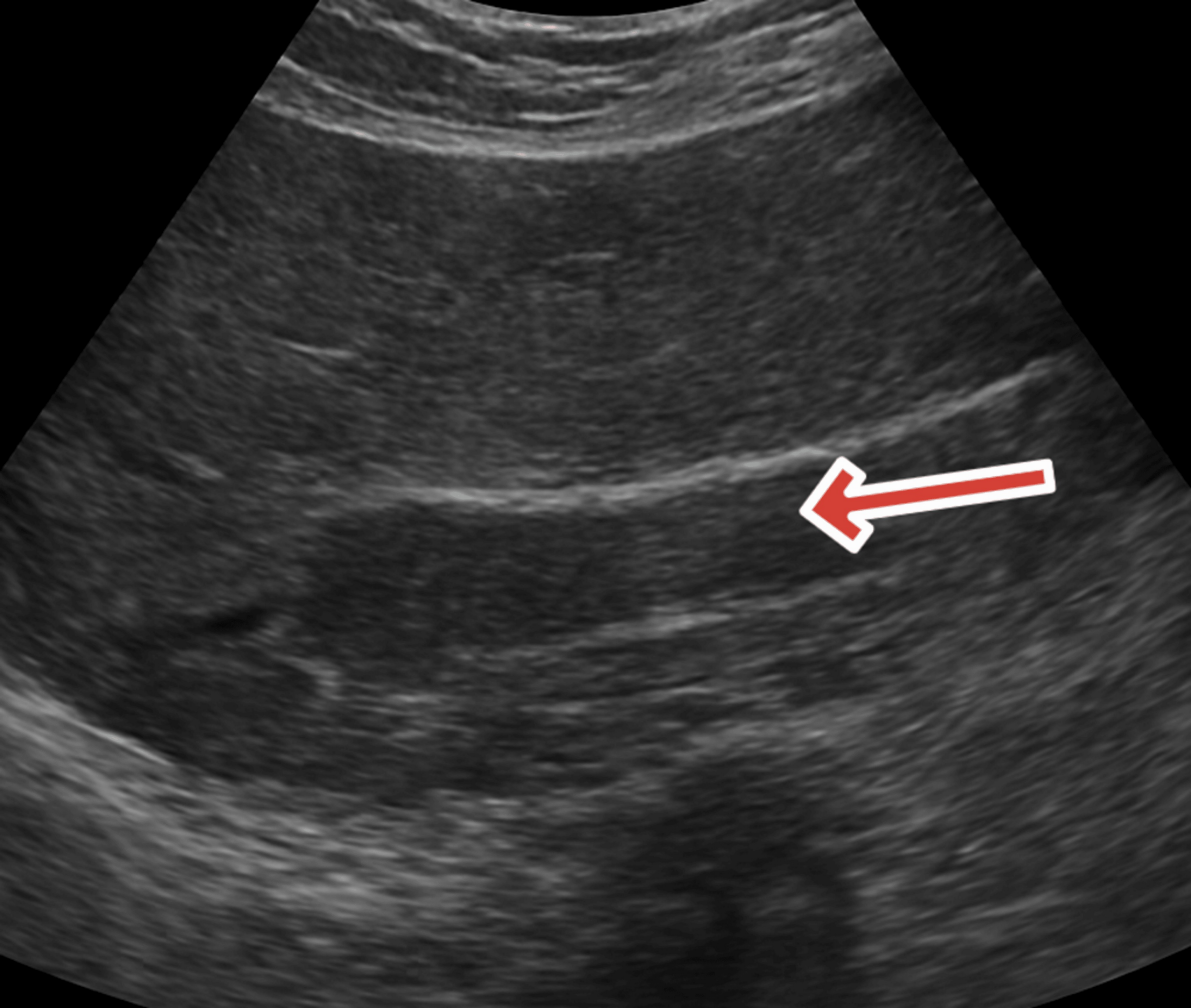
Abdomen ultrasound showing convalescent phase pancreatitis (Jan 02, 2025) The red arrow shows the subsiding and resolving phase of pancreatic tissue.

Over the next several weeks, the patient underwent three sessions of endoscopic necrosectomy via the LAMS tract to mechanically remove devitalized pancreatic tissue, each spaced several days apart to allow for interval liquefaction and safer debridement. His symptoms steadily improved, inflammatory markers normalized, and enteral nutrition was resumed. On November 29, 2024, a follow-up contrast-enhanced CT abdomen revealed residual pancreatic necrosis with the LAMS in situ, pneumobilia indicating biliary-enteric communication, and resolving peripancreatic inflammation. By January 2, 2025, a repeat ultrasound confirmed complete resolution of the walled-off collections and pseudocysts, with only mild peripancreatic fat stranding consistent with convalescent-phase pancreatitis as shown in Figure 5.

With careful outpatient monitoring, the patient was sent home in stable condition. At his most recent clinic visit, he remained asymptomatic, had regained his baseline functional status, and had no recurrence of pain, jaundice, or fever. Table 1 summarizes the chronological clinical course, interventions, and outcomes of this man with severe necrotizing biliary pancreatitis, from presentation to complete recovery.

**Table 1:** Clinical timeline of severe necrotizing biliary pancreatitis ↑ = Elevated, GB = Gallbladder, CBD = Common Bile Duct, USG = Ultrasound, LFTs = Liver Function Tests, ERCP = Endoscopic Retrograde Cholangiopancreatography, Fr = French (catheter size unit), CT = Computed Tomography, EUS = Endoscopic Ultrasound, LAMS = Lumen-Apposing Metal Stent, GI = Gastrointestinal, IV = Intravenous

Date / Timeline	Event & Findings	Intervention / Management	Outcome / Notes
Oct 21, 2024	Severe epigastric pain radiating to back with vomiting; labs: ↑amylase, ↑lipase, leukocytosis; LFTs cholestatic pattern. USG: Distended GB with multiple calculi, CBD 12 mm with 6 mm stone.	Urgent ERCP: sphincterotomy, balloon extraction, 7 Fr × 10 cm plastic biliary stent placement.	Initial stabilization.
Oct 22–23, 2024	Post-ERCP: Persistent pain, worsening systemic inflammation.	IV fluids, antibiotics, analgesia, monitoring.	Initial response, then deterioration.
Oct 24, 2024	High-grade fever (106°F), clinical decline. Contrast-enhanced CT: diffuse pancreatic enlargement, patchy necrosis, small peripancreatic necrotic collection.	Supportive management, IV hydration, escalation of antibiotics, parenteral nutrition.	Modified CT Severity Index = 8 (severe pancreatitis).
Nov 4, 2024	Repeat USG: Two enlarging peripancreatic fluid collections (84 mL anterior to body, 9.9 × 4.29 cm near tail). Persistent sepsis.	Multidisciplinary team decision for endoscopic drainage.	Planned EUS-guided drainage.
Nov 5–10, 2024	EUS-guided cystogastrostomy with LAMS placement. Complication: acute upper GI bleed. Cultures grew *Klebsiella*, *Enterobacter*, *Enterococcus faecium*.	Hemostasis achieved endoscopically; IV antibiotics tailored to sensitivities.	Source control and stabilization.
Nov 10–29, 2024	Ongoing infected necrosis with sepsis.	Three sessions of endoscopic necrosectomy via LAMS tract.	Gradual clinical improvement, pain reduction, normalization of labs.
Nov 29, 2024	Follow-up contrast-enhanced CT: residual pancreatic necrosis, LAMS in situ, pneumobilia, resolving inflammation.	Continued supportive care and monitoring.	Stable improvement.
Jan 2, 2025	Follow-up USG: Resolution of walled-off collections and pseudocysts; mild peripancreatic fat stranding.	Step-down care, oral feeding, outpatient monitoring.	Convalescent-phase pancreatitis.
Jan 2025 onward	Outpatient follow-up.	Conservative management, monitoring, dietary advice.	Complete recovery; asymptomatic, no recurrence.

Table 2 shows a progressive rise in leukocytosis, CRP, and procalcitonin correlating with clinical worsening (Oct 24), followed by a sustained decline after source control and endoscopic therapy.

**Table 2:** Trends in blood investigations and inflammatory markers

Date	Phase	WBC (×10⁹/L)	CRP (mg/L)	Procalcitonin (ng/mL)	Amylase (U/L)	Lipase (U/L)	Remarks
Oct 21, 2024	Baseline / Onset	17.5	92	1.8	1260	1840	Acute biliary pancreatitis
Oct 24, 2024	Clinical Deterioration	22.3	248	6.4	620	990	Severe necrotizing phase
Nov 4, 2024	Persistent Sepsis	19.8	210	5.2	310	460	Infected necrosis
Nov 15, 2024	Post-Intervention	13.6	84	0.9	190	240	Improvement after necrosectomy
Jan 2, 2025	Recovery	8.9	14	<0.2	120	140	Resolution and convalescence

## Discussion

This case accentuates the uncommon and clinically meaningful concurrence of infected pancreatic necrosis (IPN) and acute upper gastrointestinal bleeding (UGIB) during endoscopic treatment with LAMS. Although both conditions are separately known in SAP, their coexistence during the same therapeutic event is rarely reported. It emphasizes the timing, technique, and interdisciplinarity of managing complex pancreatitis. This report’s educational relevance and novelty illustrate how a contemporary step-up approach, while very effective in source control, can be confounded by vascular occurrences that necessitate prompt identification and management.

The management of IPN has shifted over the past two decades from open necrosectomy to minimally invasive approaches [8]. Landmark trials such as the PANTER study established that step-up strategies combining percutaneous or endoscopic drainage followed by minimally invasive necrosectomy reduce morbidity and long-term complications compared with primary open surgery [11]. Subsequent evidence has favored EUS-guided drainage as the modality of choice where expertise can be utilized due to its potential to offer direct transluminal access, allow for repeated necrosectomy, and prevent external fistulae [11,12]. LAMS has further optimized technical and clinical success with high collection resolution rates in various series reported [7,8]. Nonetheless, vascular complications are a well-established but underemphasized risk, with bleeding rates of 5% to 15% in reported cohorts [11,12]. Nonetheless, vascular complications are a well-established but underemphasized risk, with bleeding rates of 5% to 15% in reported cohorts [11,12]. Described mechanisms involve direct vessel damage at tract creation, stent flange delayed erosion into overlying vasculature, and rupture of undocumented pseudoaneurysms. In our patient, the bleeding was characterized by its immediate onset during the cystogastrostomy tract creation. This acute timing, coupled with the absence of a pre-procedural pseudoaneurysm on recent cross-sectional imaging, makes iatrogenic direct vessel injury the most probable mechanism [11]. Catastrophic splenic or gastroduodenal artery bleeding following LAMS deployment has been reported in case reports, often necessitating emergency embolization or surgery [11]. In the case at hand, intraprocedural hemorrhage was controlled endoscopically [12]. Nonetheless, the experience accentuates the inherent vascular hazards of the procedure, mainly when intervention is undertaken before a mature wall’s establishment [12].

Our patient’s course demonstrates the fundamental dilemma in managing IPN: balancing the benefits of early intervention against the risks of premature drainage. While guidelines generally recommend deferral of treatment until walled-off necrosis is present (≥4 weeks), refractory sepsis, increasing collections, or threatened organ dysfunction generally require earlier intervention. This patient’s refractory fever, leukocytosis, and radiographic deterioration despite maximum conservative management necessitated immediate intervention, even when the collections were not yet matured. Onset of hemorrhage during cystogastrostomy emphasizes the cost of this unavoidable departure from ideal timing. The microbiologic findings in this case, including *Klebsiella pneumoniae*, *Enterobacter* species, and *Enterococcus faecium*, reflect the polymicrobial etiology of contaminated necrosis and agree with published series with gut-derived flora predominance [12]. The findings reaffirm that antimicrobial treatment must be tailored to culture data and cannot replace timely source control. It poses the important question of whether pre-procedure computed tomography angiography (CTA) or Doppler-EUS must be required to detect high-risk vascular anatomy or occult pseudoaneurysms before early intervention. Though not practiced by everyone, more and more literature favors such imaging as an important risk-reduction tool, particularly in unstable patients needing urgent drainage.

The microbiologic results in this case, such as *Klebsiella pneumoniae*, *Enterobacter* species, and *Enterococcus faecium*, indicate the polymicrobial nature of contaminated necrosis and are consistent with published series with a predominance of gut-derived flora. The findings reinforce that antimicrobial therapy needs to be adjusted to culture information and cannot replace timely source control. The successful resolution of infection in this case required both targeted antibiotics and staged necrosectomies, exemplifying the complementary relationship between pharmacologic and mechanical strategies.

Several important strengths and limitations deserve emphasis. Strengths include the comprehensive documentation of the clinical course, detailed description of procedural events, and demonstration of coordinated use of ERCP, EUS-guided drainage, LAMS placement, and staged necrosectomy within a multidisciplinary framework. Limitations are inherent in a single-patient case report, prohibiting generalization, and the lack of vascular imaging before cystogastrostomy potentially has foretold or averted hemorrhage. Additionally, while medium-term follow-up confirmed resolution of necrosis, longer follow-up would provide greater assurance regarding recurrence, stent-related adverse events, and pancreatic exocrine or endocrine sequelae. The clinical implications of this case are significant. First, IPN and UGIB are rare combinations that are life-threatening, so clinicians must expect complications from drainage. Second, multidisciplinary planning with gastroenterology, interventional radiology, and surgery from the very beginning is essential to successful outcomes. Third, our case highlights that in necrotizing pancreatitis, the line between a procedure-related bleed and a natural disease complication is often blurred. We hypothesize that the procedure can provoke hemorrhage from friable, inflammation-weakened vessels, even without a macroscopic pseudoaneurysm. Therefore, pre-procedural CTA or Doppler-EUS should be standard to identify high-risk vasculature and better stratify the timing and approach of drainage, potentially preventing these hybrid complications. Lastly, although LAMS is still an effective tool, it needs to be used with sensitivity to its particular risk, and adjunctive treatment such as coaxial plastic stents or early stent removal should be contemplated in certain situations.

Finally, it is clear that despite the age of highly specialized endoscopic therapy, AP complicated by infected necrosis remains a condition requiring case-specific application of the step-up strategy, careful risk estimation, and preparedness for the treatment of vascular complications. The rare concomitant presentation of IPN and intraprocedural bleeding highlights the irreplaceable nature of prompt multidisciplinary planning and vigorous vascular evaluation. Critically reviewing this case, practitioners can better predict risks, streamline decision-making, and optimize outcomes in managing severe necrotizing pancreatitis.

## Conclusions

This case clearly demonstrates a life-threatening complication in the contemporary management paradigm for SAP: hemorrhagic shock with endoscopic drainage of infected necrosis. It emphasizes that although the step-up approach and LAMS have transformed care, they pose distinctive risks, especially when intervention is precipitated before collections are mature. The urgency of source control in a septic patient must be carefully balanced against the heightened risk of violating immature, vascular-rich tissues. The pivotal lesson from this case is the non-negotiable imperative for comprehensive vascular assessment before endoscopic intervention. Anytime clinical stability permits, pre-procedure CTA or Doppler-EUS should be made an immediate standard of care to detect pseudoaneurysms and characterize local vasculature, thus avoiding this calamitous risk. In addition, the successful outcome relied on instant access to multidisciplinary skills, concluding that treating complicated pancreatitis is an interdisciplinary effort among endoscopists, interventional radiologists, and surgeons.

Finally, this report confirms that technological progress does not diminish risk but alters its face. It is an important reminder that the successful use of advanced tools such as LAMS depends on careful planning, foresight in anticipating problems, and responsive decision-making. This experience underpins the case for standardizing vascular imaging protocols and could fine-tune guidelines on when and how to safely perform endoscopic necrosectomy in high-risk cases in the future.
